# AXZ viewer: a web application to visualize unprocessed AFM-IR data

**DOI:** 10.1093/bioinformatics/btaf513

**Published:** 2025-09-19

**Authors:** Wouter Duverger, Georg Ramer, Nikolaos Louros, Joost Schymkowitz, Frederic Rousseau

**Affiliations:** Switch Laboratory, VIB-KU Leuven Center for Brain and Disease Research, Leuven 3000, Belgium; Switch Laboratory, Department of Cellular and Molecular Medicine, KU Leuven, Leuven 3000, Belgium; Institute of Chemical Technologies and Analytics, TU Wien, Vienna 1060, Austria; Center for Alzheimer’s and Neurodegenerative Diseases, Peter O’Donnell Jr. Brain Institute, University of Texas Southwestern Medical Center, Dallas, TX 75390, United States; Department of Biophysics, University of Texas Southwestern Medical Center, Dallas, TX 75390, United States; Switch Laboratory, VIB-KU Leuven Center for Brain and Disease Research, Leuven 3000, Belgium; Switch Laboratory, Department of Cellular and Molecular Medicine, KU Leuven, Leuven 3000, Belgium; Switch Laboratory, VIB-KU Leuven Center for Brain and Disease Research, Leuven 3000, Belgium; Switch Laboratory, Department of Cellular and Molecular Medicine, KU Leuven, Leuven 3000, Belgium

## Abstract

**Motivation:**

Atomic Force Microscopy-based Infrared spectroscopy (AFM-IR) is a novel and innovative method for label-free high-resolution structural biology. However, the nature of the data files generated by AFM-IR instruments precludes investigation by conventional open-source scientific image analysis software suites. As a result, reporting of AFM-IR datasets is not standardized and the data itself is difficult to audit.

**Results:**

We have developed a web application that allows anyone to open, review, and audit raw AFM-IR data files easily and without deep knowledge of the method. It also exposes all metadata recorded by the microscope at the time of measurement. The web application is based on a Python package that supports custom data analyses within the scientific Python ecosystem. This tool provides an accessible, transparent solution for AFM-IR data review, with the potential to support reproducibility and standardization in AFM-IR research and encourage wider adoption of this innovative spectroscopy method.

**Availability and implementation:**

The web app is hosted at https://anasys-python-tools-gui.streamlit.app. Its source code is listed at https://github.com/wduverger/anasys-python-tools-gui. The underlying Python package is available at https://github.com/GeorgRamer/anasys-python-tools and can be installed using pip.

## 1 Introduction

Infrared (IR) absorption spectroscopy is a workhorse in modern life sciences and beyond. It is a collection of physicochemical methods that probe the vibrational energy levels of chemical bonds between atoms and is therefore sensitive to the chemical composition and structural properties of molecules in a sample, such as protein conformation (Bec *et al.* 2020). Although traditional IR microscopy is non-invasive, fast, and label-free, it offers insufficient resolution for nanoscale structures due to wavelength limitations and light diffraction (Poli *et al.* 2012). Recently, a number of innovative Atomic Force Microscopy (AFM) methods have been developed that increase the resolution by two to three orders of magnitude, such as scanning near-field optical microscopy (SNOM) and Atomic Force Microscopy-based Infrared spectroscopy (AFM-IR) (Dazzi and Prater 2017), by using a sharp AFM probe to achieve highly localized signal detection.

In contrast to SNOM, where light scattered by the sample is interrogated to study its complex refraction coefficient, the AFM-IR readout is absorption-based and mechanical: the probe measures the thermal expansion of a sample upon absorption of the light from an infrared laser. Therefore, AFM-IR spectra conserve proportionality to well-known Fourier Transform Infrared (FTIR) spectra (Dazzi *et al.* 2010, Ramer *et al.* 2017, Quaroni 2020). The high spatial resolution of AFM-IR and its potential to discriminate small structural and chemical variations in a sample means the method has found its way to many domains of life and material sciences.

AFM-IR has found applications in the mapping of distributions of enzymes, lipids, or drug molecules in whole cells (Pancani *et al.* 2018, Dos Santos *et al.* 2020, Deniset-Besseau *et al.* 2021, Liu *et al.* 2021). The pathophysiology of the infection of red blood cells by malaria parasites has been studied with AFM-IR (Perez-Guaita *et al.* 2018), as well as breast cancer (Petay *et al.* 2022) and the effects of irradiation on prostate cancer cells (Roman *et al.* 2019). It has been possible to reproduce Giemsa banding (staining of condensed versus transcriptionally accessible chromosomal DNA) in a single-molecule label-free manner (Lipiec *et al.* 2019). Under perfect conditions, AFM-IR is capable of detecting infrared spectra of protein fibrils and even single proteins (Ruggeri *et al.* 2019, Waeytens *et al.* 2021). Furthermore, nanospectroscopy holds promise in fields beyond biology. For example, it can interrogate the material properties of polymers and blends in terms of their nanoscale composition and structural properties (Tseng *et al.* 2022, Brütting *et al.* 2023), with applications in semiconductors (Lo *et al.* 2016), perovskite photovoltaics (Yuan *et al.* 2015, Tseng *et al.* 2022) and even asteroids (Dartois *et al.* 2023).

The advanced nature of AFM-IR microscopy causes three problems. First, the data files generated by commercial microscopes (such as Bruker nanoIR instruments) are of a particular format that few programs can read. There are solutions, such as Gwyddion (Nečas and Klapetek 2012), Mountains (Digital Surf, France), or Quasar (Toplak *et al.* 2021), but these programs are closed-source or discard valuable metadata since they are not designed specifically for AFM-IR, see Table 1. Moreover, neither offers the capability to quickly inspect files via a web application that does not require additional software installation. Another popular image viewer, ImageJ, does not support AFM-IR files at all, nor do its extensions.

**Table 1.** Comparison between software suites.

	This paper	Gwyddion	Mountains	Anasys Studio	Quasar
Open Source	Yes	Yes	No	No	Yes
Designed for nanoIR	Yes	Yes	No	No	Yes
Spectra	Yes	No	Yes	Yes	No
Images	Yes	Yes	Yes	Yes	Yes
Auditable data analysis	Yes (Python)	Yes, if using scripts	Yes	No	Yes
Can be used without installing	Yes	No, and involved (docker is easiest)	No	No, and only available on instrument	No
Automatic QC	Yes	No	No	No	No

Second, AFM-IR measurements are prone to a range of technical issues that can lead to artefacts if not addressed. Important considerations include:

- minimizing probe oscillations and absolute deflection to <±0.01 V from the setpoint, by using slow scanning speeds,
- maintaining the IR pulse frequency at the mechanical resonance modes of the sample-probe system via a properly configured phase-locked-loop (PLL), which tracks the phase difference between IR pulses and the oscillatory thermal expansion of the sample,
- accounting for the strong and wavelength-dependent absorption of mid-IR light by atmospheric water vapour, and
- avoiding sample scratching and melting.

The third challenge, given the sensitivity of AFM-IR data to instrument settings and environmental factors, is to establish widely accepted data quality standards. This is challenging, but crucial for cross-study comparisons and reproducibility (Burnham *et al.* 2023, Dos Santos *et al.* 2023). In previous work, we have outlined a protocol which avoids many of the aforementioned technical issues in order to be able to quantitatively compare AFM-IR data across samples and experimental sessions (Duverger *et al.* 2024).

Here, we publish a freely accessible web application for rapid visualization and quality assessment of raw AFM-IR data, even if further consensus in the field is essential to establish universal data quality and reporting criteria. The application is optimized for preliminary data review and does not support complex multivariate analyses directly within the web interface. Therefore, we also publish a Python library for further analysis of this data using the extensive ecosystem of scientific Python packages.

## 2 Availability and implementation

The web application (version 1.0) is accessible at https://anasys-python-tools-gui.streamlit.app/. It wraps the Python library described later and is implemented using the Streamlit framework, which is compatible with recent versions of all major browsers. The source code for the web app is accessible at https://github.com/wduverger/anasys-python-tools-gui and https://doi.org/10.6084/m9.figshare.27991898.

The Python library at the foundation of the web app is pip-installable using “pip install anasyspythontools” and its source code is available at https://github.com/GeorgRamer/anasys-python-tools and https://doi.org/10.5281/zenodo.14447283.

It works by decompressing the .axz files generated by nanoIR systems (Bruker, Germany) and parsing the embedded XML document into a Python object containing all AFM and AFM-IR maps, optical images, and AFM-IR spectra with corresponding metadata. For ease of use, these data structures can be converted to xarray structures (Hoyer and Hamman 2017).

## 3 Features

Upon accessing the web application, the user can easily upload their own data or choose to view an example file. This file is included in the application’s GitHub repository. The data was generated as in our previous work (Duverger *et al.* 2024). Once a file is selected, four views are accessible through the sidebar: Optical Images, Map Overview, Map QC and Spectra. A demonstration video is also available on the main page to guide users through the application’s features.

**Figure 1.**
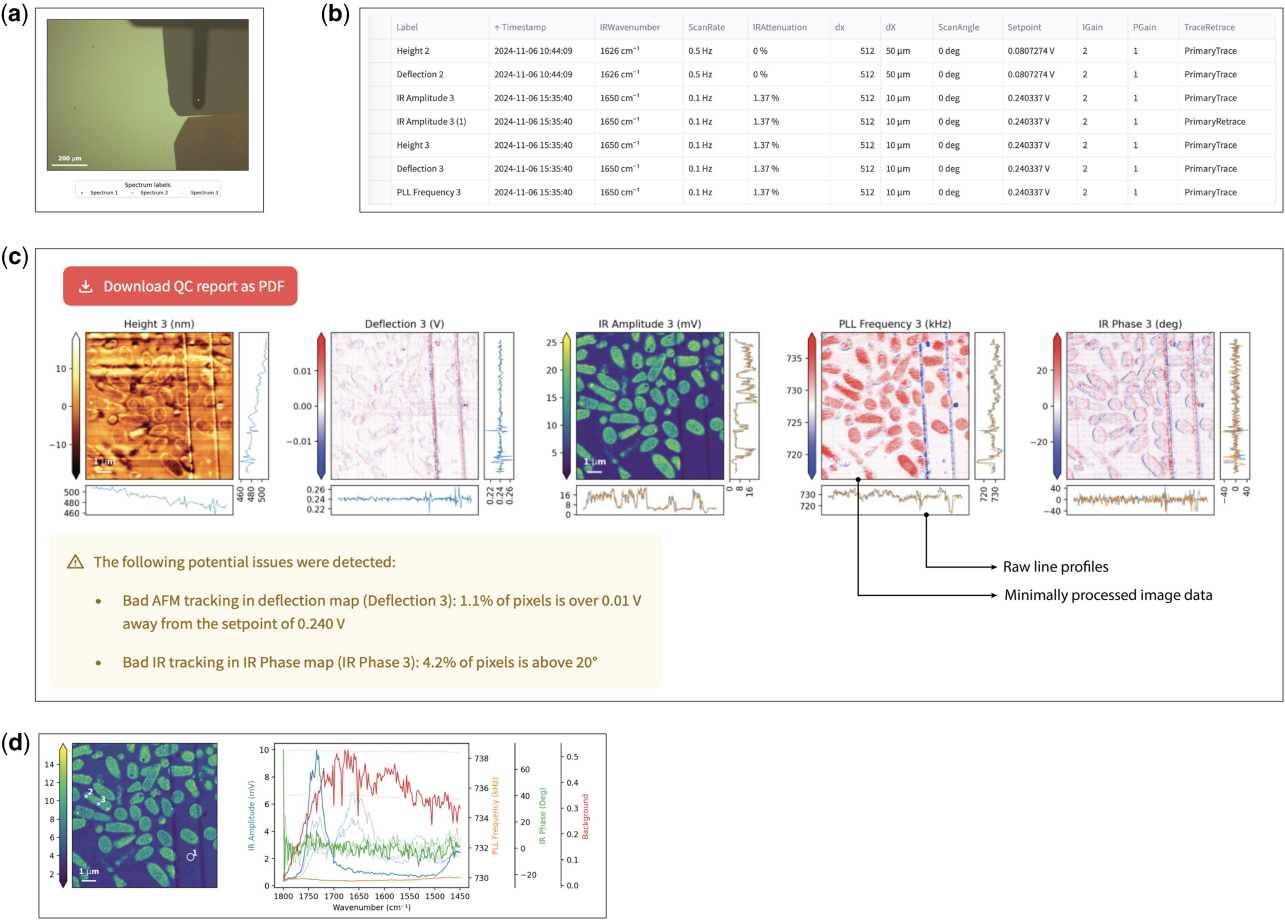
Features of the web application. (a) Localization of maps (not shown) or spectra (dots) within optical reference images. This can aid future measurements on the same sample or provide important context. (b) A subset of metadata properties of IR maps that are easily accessible in the Map Overview page. Users can select which properties to show or hide. (c) AFM-IR quality control view of a set of simultaneously recorded maps, each with a specific colourmap for clarity. Each image is accompanied by a colourbar to interpret image values, as well as line profiles showing trace (blue) and retrace (orange) data, if both were recorded. Note that height maps and deflection maps are minimally processed (linear fit and setpoint subtraction, respectively), while line profiles still show the raw data. (d) Data quality control of AFM-IR spectra, with spatial reference on the left and all available, unprocessed data channels on the right. Spectrum **1** is highlighted by a circle in the spatial reference and is indicated by bold lines in the spectral data on the right.

The first view, Optical Images (Fig. 1a), provides a collection of all optical images collected in an AFM-IR file and shows the colocalization of AFM-IR maps and spectra. The Map Overview screen displays those AFM-IR maps in greater detail. AFM-IR maps can be compared to each other and a metadata table can be displayed, detailing all experimental parameters recorded by the microscope at the time of measurement.

While the Map Overview screen can provide an overview of all maps collected during the experiment and their metadata (Fig. 1b), the Map QC page (Fig. 1c) serves a different purpose. It displays sets of maps collected at the same time, in such a way that the raw data is preserved as much as possible. There is an option to download a PDF report of these figures. In order to flag potential quality issues with a dataset, a heuristic algorithm is included to alert users to some of the most common problems, such as apparent data processing (when height, deflection, or PLL images seem flattened prior to saving, this results in data values very close to zero), and indications of flawed AFM tracking (deflection values more than 0.01 V away from the setpoint) or PLL tracking (reflected by IR phase values over 20° away from the setpoint or saturation of the PLL loop).

Finally, the Spectra tab (Fig. 1d) provides an overview of all recorded spectral data without any processing. The user can select to show all spectra together or one-by-one. The application also exposes spectrum metadata and localization information. By default, all available data channels (IR Amplitude, IR Phase, PLL frequency, …) are displayed, but irrelevant ones can easily be hidden. Spectra can be exported as a CSV file, and, as in the Map QC screen, a PDF export option is available.

The foundation of the graphical user interface is a pip-installable Python package parsing the data in nanoIR data files (with .axz extension) into numpy or xarray objects, allowing for advanced data analysis and visualization procedures leveraging the scientific Python ecosystem (Ramer *et al.* 2023, Duverger *et al.* 2024). The advantage of analyses performed this way is that they can be published alongside the raw data, making the analysis and its conclusions more easily reviewable and extensible.

The primary function of the package, anasyspythontools.read(filepath), takes a file path to a .axz file as an argument and returns an object of type AnasysDoc. This object contains optical images, AFM-IR maps, and spectra as the dictionaries AnasysDoc.Images, AnasysDoc.HeightMaps, and AnasysDoc.RenderedSpectra respectively. Each dictionary contains objects that store both the experimental data (through the property SampleBase64), and metadata (through its other properties). These property names are chosen to match the underlying XML representation. Additionally, images, maps, and spectra can be converted to xarray objects using functions in anasyspythontools.export module. For usage examples, please refer to the Jupyter notebook located in the examples folder of the package’s GitHub repository.

To ensure the usefulness of this application, we solicited community feedback from two independent research groups and performed benchmarking of its most critical components. Both research groups found this application to be a valuable addition to the field and suggested some minor enhancements, such as the possibility to export spectra as a CSV file, discussed above. The tool has already been integrated into internal workflows for rapid and interactive visualization of AFM-IR data, while away from the instrument. It also guides data analysis decisions, which are then performed using the associated Python library. In fact, similar quality assessments performed with this tool have appeared in the supplementary materials of previously published studies (Duverger *et al.* 2024). These instances demonstrate the tool’s utility in streamlining data evaluation and supporting reproducible research.

Regarding the application’s performance, the included example file has a size of 26 MB. The application accepts files up to 200 MB, while the instrument’s measurement software prohibits files above 50 MB. On the example file, upload takes around 15 s, depending on the connection, and generating a full map QC report takes another 10 s. However, a caching system is in place to speed up subsequent page views. Because the cache is saved to Streamlit’s session state, it is user-specific and purged on disconnection. At this point, the bottleneck is the repeated downloading of the image data from the Streamlit servers to the local viewer. Users who need to view many files and wish a faster application can easily download and install the application locally, following the instructions provided in the GitHub repository. Finally, since the public version is hosted on Streamlit Cloud, it will be shut down after 24 h of inactivity. Booting it up takes around 40 s.

## 4 Discussion

We report the development of a Python package to parse AFM-IR data files enabling advanced data analysis, and a web application for viewing their contents without the need for installation of any software. By providing accessible tools for data review, we aim to enable both experienced and new users to critically assess the quality of AFM-IR data. Our tool supports the broader adoption of open data practices, promoting the publication of raw data and code to facilitate reproducible research across the field. The web application intentionally has a limited scope, with the goal of making data visualization as easy and as fast as possible for users and non-users alike. Since data analysis is much more application-specific, we prefer to use the Python package, which can easily interface with the broad scientific ecosystem of Python libraries. As such, we foresee limited future expansions, except to improve compatibility with additional file formats, such as those generated by the newest line of commercially available nanoIR instruments (such as the IconIR series of instruments, by Bruker), or to maintain alignment with evolving data quality standards in the AFM-IR community.

## Data Availability

The data underlying this article are available in GitHub, at https://github.com/wduverger/anasys-python-tools-gui and https://doi.org/10.6084/m9.figshare.27991898.
